# An opportunistic observational study of hospital switchboard hold messages, hold music and time spent waiting

**DOI:** 10.1016/j.fhj.2025.100224

**Published:** 2025-01-22

**Authors:** Jordan P. Skittrall, Mary D. Fortune

**Affiliations:** a Department of Pathology, University of Cambridge, Addenbrooke's Hospital, Cambridge CB2 0QQ, United Kingdom; b Department of Public Health and Primary Care, University of Cambridge, Forvie Site, Cambridge CB2 0SR, United Kingdom

**Keywords:** Switchboard services, Hospital communication systems, Communicable disease control, Patient visitors

## Abstract

Numerous callers contact hospital switchboards, many repeatedly. Recorded switchboard messages have broad reach. We sampled outgoing calls from our regional infectious diseases service to other hospital switchboards over a 3-month period. Wait times varied substantially. Two calls were cut off unanswered. Recorded messages commonly covered infection control precautions for visitors, visiting rules, and guidance regarding calling for medical advice. At least one message was out of date. Another advised unwell people not to visit the hospital. We followed up our sample with freedom of information requests, focusing on call volume data and policies regarding switchboard systems and messaging. Many policies were informal. Overall, the user experience varied substantially between hospitals. Messaging policies appeared to have little regard to messages’ relevance, utility and impact. Calling a hospital switchboard is a common, non-biomedical task that would benefit from substantial effort to establish and promote best practice.

## Highlights

• Recorded message content used by hospital switchboards seldom follows best practice.
• Review of recorded messages is irregular and informal.
• Hold music selection by hospitals follows a path of least resistance.

## Graphical abstract

**Figure:**
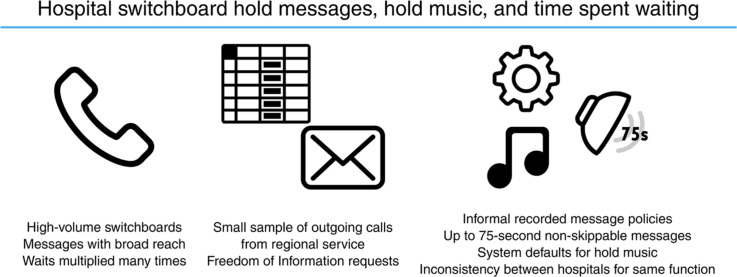

## Introduction

Hospital switchboards are contacted by an undifferentiated variety of callers. Callers include relatives seeking visiting information or status updates, members of the public seeking advice, clinicians trying to convey information, and callers involved in support of a large organisation. Switchboard response times have previously been reported as variable, with recorded infection control messages associated with increasing delays to first response but not necessarily reflecting community prevalence of illness or improving infection control outcomes.^1^, ^2^, ^3^ Automated call-handling systems have been reported to be slower than human call-handlers.^2^

Hold music has long been recognised as confirming a call remains active, and hence reducing call abandonment.^4^ The type of music played has been shown to influence caller aggression.^5^ Information about wait times and queue position reduces overestimation; those thinking they have waited less long are more satisfied.^6^

We aimed to describe contributors to variability in hospital switchboard response times, particularly from policies relating to staffing and recorded messages. We also aimed to describe factors influencing message and hold music selection.

## Methods

From November 2023 to February 2024, we opportunistically documented outgoing calls to hospital switchboards from our infectious diseases service, based in the East of England, as part of the routine functioning of the tertiary referral service. We documented details and duration (to nearest 5 seconds) of automated messages, type of initial navigation (voice-activated, keypad options or extension dialling, or direct-to-human), time to first caller input (including answer by call-handler), name of hold music, and interruptions during hold music including details of position in queue. We identified hold music using the Shazam app (Apple Inc). If, within a call, we needed to redial the switchboard, we considered the first attempt only.

We sent freedom of information requests to all hospitals included, querying switchboard call volumes and call handling policies.

## Results

### Summary of findings

We collected data on 55 calls to hospital switchboards from November 2023 to February 2024. Thirteen hospitals, representing 11 distinct NHS trusts, were contacted. Table 1 summarises waiting times, switchboard system types, hold music use and monthly call volumes.

**Table 1:** Summary of calls to hospital switchboards.

Each row contains summary data for one hospital. The hospitals in the two rows marked on the left with a single solid black line are in the same trust. The hospitals in the two rows marked on the left with a double solid black line are in the same trust. Where more than one system type is recorded for a switchboard, this is because different call handling systems were encountered during different calls. The hold music column only details whether music was heard prior to first answer or first required caller input.

### Time spent waiting

**Fig. 1:**
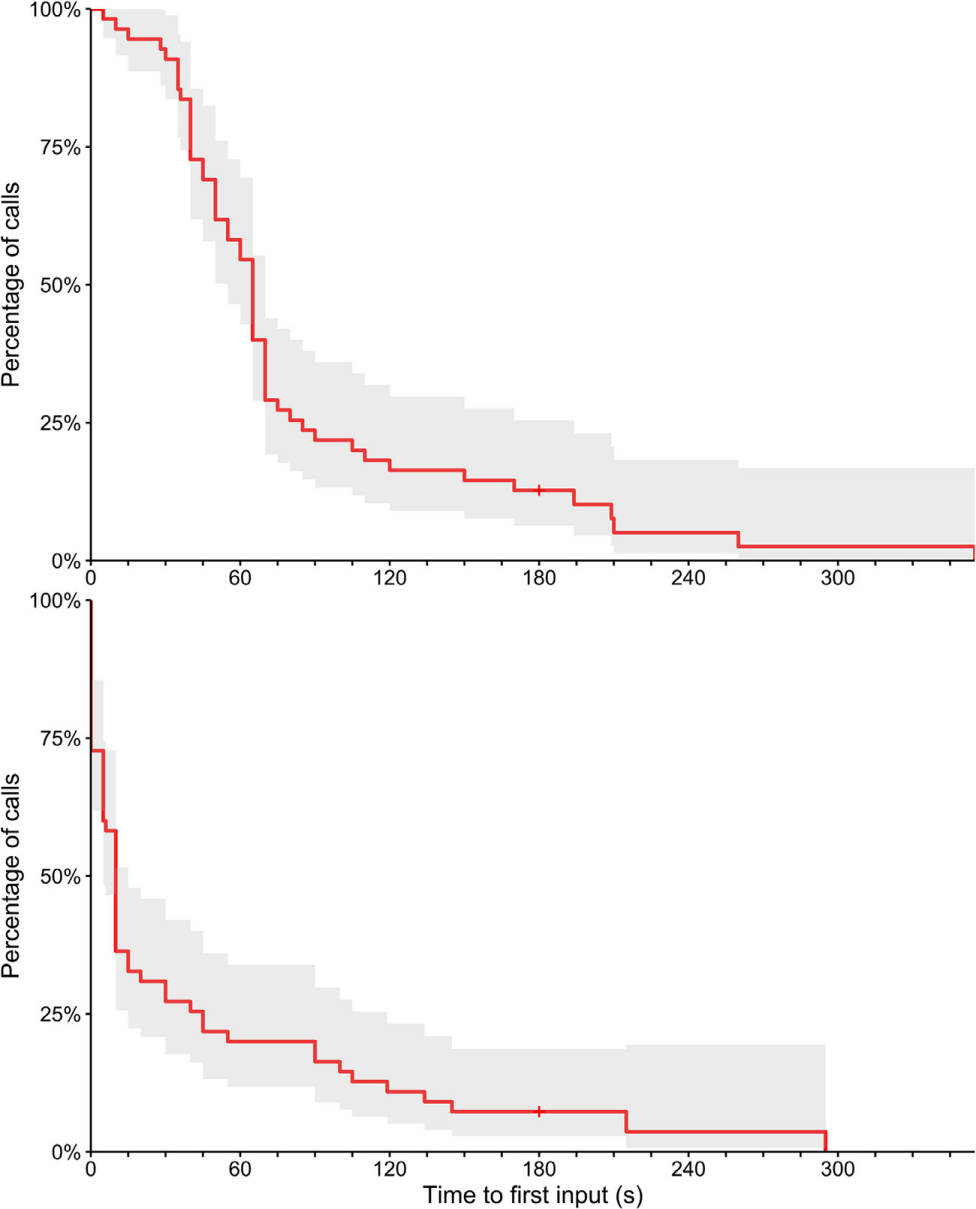
Top: Total time until first answer or required caller input, including time spent listening to non-skippable recorded messages, measured in seconds; dropped calls were treated as censored at the time of dropping. Bottom: Total time until first answer or required caller input excluding time spent listening to non-skippable recorded messages, measured in seconds; dropped calls were treated as censored at the time of dropping.

Total time between finishing dialling the switchboard number and first caller input/answer by human switchboard operator varied from <5 s (answered by a human on the first ring) to 355 s, with a median of 65 s. Fig. 1 (top panel) plots this waiting time for all calls.

Two calls were dropped without answer after 3 min with no messages heard; the caller then heard a busy tone or a message ‘there is no reply’. A third hospital’s call handling system dropped a call after 6 min on hold awaiting a response from a ward, without redirection back to the switchboard.

Twelve of the 13 switchboards played non-skippable recorded messages of between 20 s and 75 s. Fig. 1 (bottom panel) shows the waiting time distribution after excluding these non-skippable messages.

### Content of recorded messages

The content of non-skippable messages varied, even between hospitals in the same trust. Most concerned visiting rules (6/13 hospitals), visiting with/after symptoms of diarrhoea (6/13 hospitals), visiting with respiratory/COVID-19 symptoms (7/13 hospitals), and rules regarding masking (2/13 hospitals), or asked callers not to contact the hospital directly for medical advice (3/13 hospitals).

Some messages contained out-of-date information (for instance, one warning of ‘current’ industrial action that had finished). Of particular concern was the message ‘Please be aware that we remain highly vigilant to the spread of infections or illnesses, so if you are in any way unwell, please do not visit our hospitals’, which could be misconstrued as dissuading callers from seeking appropriate medical care.

### Switchboard systems

Of the 13 hospitals included, three always placed callers on hold until a human operator answered, four usually allowed callers to bypass a human operator by dialling an extension, and six usually sent callers to a voice-activated automated system to direct calls.

Five different hold music tracks were heard while awaiting a response from switchboards, and an additional four hold music tracks were heard at other times during calls. All hold music identified was classical genre or specifically composed for hold tracks; where it was possible to determine this, music was royalty-free. Hospitals not playing hold music played a ringing tone. Four hospitals that played hold music interrupted it with other messages during the time on hold, but none of them consistently.

### Trust switchboard policies

Ten out of the 11 NHS trusts sent questionnaires replied. Among responding trusts, daily call volumes reported had a median of approximately 1,200 calls (interquartile range 900–1,800), corresponding to a monthly median of approximately 38,000 (interquartile range 28,000–54,000). One trust’s staffing policy accounted for varying call volumes over the day; the remainder had flat minimum staffing levels and/or no written policy.

Most trusts identified executives/their offices and/or infection control teams as responsible for deciding non-skippable messages played to callers. No trust reported a written policy for deciding or reviewing message content.

Trusts reporting policies to decide hold music either reported using the system default (five trusts) or named a royalty-free track (one trust).

Four out of ten trusts gave average call response times. Three reported not recording response times; three gave replies without response times.

Most trusts gave generic responses regarding policies to handle unanswered calls, indicating that all calls should remain in the same queue until answered. One of the trusts that dropped initial calls to a switchboard did not reply to the questionnaire, and the other did not answer the question on unanswered call handling. The trust where a call put through to a ward was dropped reported that it did not have a procedure for unanswered calls, but was considering one.

## Discussion

Our small, opportunistic study suffices to demonstrate a remarkable heterogeneity in experiences of callers to hospital switchboards. The different call handling technologies in use explain some, but not many, differences. Particularly remarkable is the variety in recorded messages, and evidence gained by freedom of information requests that this variety results not from applying an evidence base in differing circumstances, but instead from informal decision-making processes. Epitomising this phenomenon is the recording containing an ambiguity interpretable as suggesting that sick people were unwelcome at the hospital. No hospital reported a policy considering their recorded messages’ length. For many members of the public, the switchboard is their first point of contact with the hospital, making the appropriateness of messages conveyed through the switchboard – especially messages delivered without direct human oversight – especially important.

The variety of users calling hospital switchboards means that recorded messages will not be relevant to all. In many cases, including our context of an infectious diseases service, callers redial the same switchboard many times. Such people will have spent many minutes repeatedly hearing the same information, much of it irrelevant to their call. Given the call volumes reported, for many hospitals the cumulative time that callers spend listening to recorded messages exceeds a full-time job. It is unclear whether benefits from hearing non-skippable messages outweigh disadvantages from the time and cost incurred.

The absolute number of dropped calls was low (2/55). However, such calls are a particular concern since the recipient cannot know what information has not been conveyed. It seems likely that dropped calls were caused by either call-handling system configurations, or system technical issues.

Hold music choice seemed to follow a path of least resistance, with the options used being those easiest to set up or cheapest. No hospital reported explicitly considering music’s effect on outcomes such as caller emotions.

There is little evidence base regarding strategies for handling calls to hospital switchboards. Our study shows the evidence that does exist is seldom applied, and a major point of contact between hospitals and the public and external professionals is lightly overseen. While the consequences of these policies for individual callers are usually slight, the volume of repetition yields substantial potential for these decisions to have a larger cumulative effect.

The experiences related correspond to a small, opportunistic, non-systematic sample. Samples from different switchboards may reflect periods of different underlying activity. While able to showcase heterogeneity in policies and experiences and to serve as a call to action, the records of calls cannot be used to make conclusive comparisons between individual hospitals. Owing to incomplete responses to freedom of information requests, it is not possible to investigate correlations between waiting times experienced in our study and variables such as call volumes. Most of the hospitals included in the study were district general hospitals and there were too few hospitals of other types to draw reliable conclusions about wait time differences between types of hospital. All switchboard operators contacted were helpful and efficient.

We acknowledge that, in some circumstances, professionals have access to telephone numbers that will either allow them to bypass hospital switchboards entirely, or will allow them to skip recorded messages. We have not investigated the utility of such workarounds – although their very presence suggests the existence of a problem to be solved. Some means of sharing such numbers are informal, and are prohibited by some NHS trusts for security reasons. Even if such workarounds were widely used by professionals, our conclusions would still apply to other categories of caller.

Hospitals should spend time optimising the experience of automated, highly repetitive events, especially ones involving the public or external professionals. For switchboard calls, hospitals should consider each aspect of user experience, including recorded messages and hold music. Hospitals should develop policies for regularly reviewing information conveyed by switchboards.

This study highlights the need for a clear evidence base, tailored to the hospital setting, regarding what constitutes effective messaging to those calling switchboards, and considering inadvertent harms from increasing caller wait time. Clearer evidence regarding the effects of differing hold music on callers to hospitals is likely to be impactful, owing to the number of people affected.

Our study specifically investigated hospital switchboards. Nonetheless, we anticipate that many of the lessons learned will be applicable to other healthcare settings, for example to call handling in general practice.

## Ethics approval and consent to participate

This study was evaluated using the NHS Health Research Authority decision making tool and was deemed not to constitute research within the NHS meaning of the term. Ethical approval is not required.

## Data availability

The data that support the findings of this study are available from the corresponding author upon reasonable request.

## Funding statement

JPS is funded by the National Institute for Health and Care Research (NIHR). No other author has received external funding for this study. The funder had no rule in the study design; in the collection, analysis and interpretation of data; in the writing of the report; or in the decision to submit the article for publication. The views expressed are those of the authors and not necessarily those of the NIHR, the NHS, or the Department of Health and Social Care.

## CRediT authorship contribution statement

**Jordan P. Skittrall:** Writing – review & editing, Writing – original draft, Visualization, Methodology, Investigation, Data curation, Conceptualization. **Mary D. Fortune:** Writing – review & editing, Visualization, Methodology, Formal analysis, Data curation.

## Declaration of competing interest

The authors declare the following financial interests/personal relationships which may be considered as potential competing interests: Jordan P. Skittrall reports financial support was provided by National Institute for Health and Care Research. If there are other authors, they declare that they have no known competing financial interests or personal relationships that could have appeared to influence the work reported in this paper.

## References

[1] Shokrollahi K., Tadiparthi S., Jayagopal S. (2008). How fast is fast enough? An audit and league table of response times of acute hospital NHS Trust switchboards in England. J R Soc Med.

[2] Ghelani R., Maclean E., Adra M. (2019). Identifying avoidable switchboard delays in England's NHS hospitals: phase one of the national SWITCH project. Acute Med.

[3] Maclean E., Ghelani R., Adra M. (2020). Recorded infection control messages delay inter-professional communication but are not associated with COVID-19 prevalence or mortality: insights from a national switchboard analysis, Clinical Medicine. J R Coll Physicians Lond.

[4] Roth M. (1993). What do your patients hear when they're put on “hold”?. J Michigan Dent Assoc.

[5] Niven K. (2015). Can music with prosocial lyrics heal the working world? A field intervention in a call center. J Appl Soc Psychol.

[6] Whiting A., Donthu N. (2009). Closing the gap between perceived and actual waiting times in a call center: results from a field study. J Serv Marketing.

